# RNNet-MST: A ResNet-50 with Multi-Scale Transformer Blocks for Pulmonary Nodule Classification and Attention-Based Localization on Chest X-Ray Images

**DOI:** 10.3390/diagnostics16101574

**Published:** 2026-05-21

**Authors:** Edrill F. Bilan, Emman T. Manduriaga, Hernando S. Salapare, Ymir M. Garcia, Khatalyn E. Mata, Rose Anna R. Banal, Imelda C. Ang, Wei-Ta Chu, Dan Michael A. Cortez

**Affiliations:** 1 College of Information Systems and Technology, Pamantasan ng Lungsod ng Maynila, Manila 1002, Philippines; efbilan2022@plm.edu.ph (E.F.B.); emmanmanduriaga@gmail.com (E.T.M.); kemata@plm.edu.ph (K.E.M.); 2 Institut de Science des Matériaux de Mulhouse (IS2M), CNRS, UHA, UMR 7361, 68057 Mulhouse, France; 3 University Research Center, Pamantasan ng Lungsod ng Maynila, Manila 1002, Philippines; 4 School of Chemical, Biological, Materials Engineering and Sciences, Mapúa University, Manila 1002, Philippines; ymgarcia@mapua.edu.ph; 5 School of Health Sciences and Nursing, Mapúa University, Makati 1205, Philippines; 6 College of Medicine, Pamantasan ng Lungsod ng Maynila, Manila 1002, Philippines; rarbanal@plm.edu.ph (R.A.R.B.); icang@plm.edu.ph (I.C.A.); 7 Department of Computer Science and Information Engineering, National Cheng Kung University, Tainan 701401, Taiwan; wtchu@gs.ncku.edu.tw

**Keywords:** RNNet-MST, pulmonary nodule detection, pulmonary nodule classification, deep learning, ResNet-50, transformers, spatial attention mechanism, chest X-ray analysis, computer-aided detection, false negative reduction

## Abstract

**Background/Objectives**: Lung cancer survival depends on early detection; however, in the Philippines, high radiologist workloads and the anatomical complexity of chest X-rays (CXRs) contribute to missed pulmonary nodules and false-negative diagnoses. This study aims to develop an enhanced deep learning model to improve nodule classification and localization sensitivity. **Methods**: We propose RNNet-MST, an extension of ResNet-50 that incorporates Multi-Scale Transformer blocks for global context modeling and a custom spatial attention mechanism for attention-based weak localization of disease-relevant regions. The model was trained and evaluated on the NODE21 chest X-ray dataset and compared with a baseline ResNet-50 using classification metrics, with attention maps used for weak localization analysis. **Results**: RNNet-MST demonstrated consistent improvements over the baseline ResNet-50 across evaluated metrics. Mean Nodule Recall improved from 88.02 ± 1.92% to 91.55 ± 1.41%, reducing false negatives. Mean Test Precision reached 90.46 ± 0.99%, and mean Nodule F1-Score improved to 90.99 ± 0.39%. On the isolated small-nodule subset, RNNet-MST achieved a 12.3% improvement in sensitivity over the baseline. **Conclusions**: The integration of multi-scale transformer features improved classification sensitivity, while the attention mechanism provided weak localization cues that aligned more closely with annotated nodule regions than the baseline. RNNet-MST shows potential as a diagnostic support tool, warranting further validation on larger and more diverse clinical datasets to reduce perceptual errors and facilitate early lung cancer detection in resource-constrained settings.

## 1. Introduction

Lung cancer remains one of the leading causes of cancer-related mortality worldwide, and its prognosis is highly dependent on early detection [1]. In the Philippine context, the burden of life-threatening diseases continues to pose a major public health concern. Recent data indicates that neoplastic diseases rank among the top five leading causes of death nationwide [2]. This highlights an urgent need for improved diagnostic approaches, particularly for lung cancer, where early identification is crucial for improving survival outcomes.

Chest radiography (CXR) is the most widely used imaging modality in clinical practice due to its accessibility, low cost, and relatively low radiation dose compared to computed tomography (CT) [3]. CXR serves primarily as a first-line screening tool. However, detecting pulmonary nodules—small growths typically less than 3 cm—on CXRs is a highly challenging task.

Consequently, CXR interpretation is highly susceptible to diagnostic limitations and false-negative findings. A prospective study by Miki et al. demonstrated that radiologists significantly tend to miss lung nodules located in anatomically complex areas, such as the bilateral hilar regions, revealing inherent limitations in human visual search behavior [4]. Further evidence from Digumarthy et al. utilized simulation-based approaches to show that even with targeted training and education, substantial diagnostic blind spots persist in complex anatomical locations [5].

While CT offers superior spatial resolution and sensitivity for nodule detection, it is considerably more expensive, delivers a substantially higher radiation dose, and requires specialized infrastructure that is often unavailable in low- and middle-income settings [3]. As a consequence, the majority of high-performing deep learning Computer-Aided Detection (CAD) systems in the literature have been developed and evaluated primarily on CT or low-dose CT (LDCT) datasets [6,7]. CXR-based nodule detection has received comparatively less attention, despite CXR remaining the predominant—and in many rural or resource-constrained facilities, the only—available imaging modality [8]. This creates a significant translational gap: state-of-the-art CT-focused models are not clinically deployable in settings where CT infrastructure is absent, yet the CXR-focused literature reports considerably lower sensitivity benchmarks. Critically, the goal of a CXR-based CAD system in such environments is not to replace CT diagnosis, but to serve as a low-cost, computationally lightweight second opinion—flagging suspicious regions that a fatigued radiologist might overlook and thereby triaging patients who warrant further investigation.

To contextualize these limitations within current real-world diagnostic practices, we conducted expert interviews with radiologists at the Lung Center of the Philippines (LCP). The interviews revealed that radiologists face a substantial daily workload, interpreting approximately 100 to 200 radiographic images alongside other imaging modalities. This intensity causes severe cognitive load and professional burnout, which are known risk factors for perceptual and interpretive errors. Furthermore, the experts noted a lack of locally developed medical imaging technologies tailored to these specific systemic constraints.

These insights reveal a critical diagnostic gap that contributes to delayed diagnoses. Given the widespread reliance on CXR screening, there is a vital need for intelligent, automated Computer-Aided Detection (CAD) systems. Deep convolutional neural networks, specifically ResNet-50, have demonstrated strong performance in extracting fine-grained features for medical imaging using residual learning [9]. While standard ResNet-50 performs well on binary classification, its limited ability to capture multi-scale features and model long-range dependencies reduces its sensitivity to small nodules [10]. Furthermore, standard deep vision models often lack the spatial attention required to isolate subtle, overlapping abnormalities [11].

To address these limitations, this study proposes RNNet-MST, an enhanced ResNet-50 architecture for pulmonary nodule classification on chest X-rays, with attention-based weak localization used to highlight disease-relevant regions. While hybrid CNN–Transformer models have been explored previously, the specific architectural novelty of the proposed RNNet-MST lies in its deep, stage-wise integration strategy paired with a dual-branch output. Standard hybrid models typically append Transformer blocks at the very end of a CNN backbone, or they rely purely on Transformer architectures with either patch tokenization (e.g., ViT [12]) or hierarchical tokenization (e.g., Swin [13]). In contrast, RNNet-MST integrates Multi-Scale Light Transformer (MST) blocks sequentially at every single stage (Stages 1 through 4) of the ResNet-50 backbone. This means that at each distinct semantic level (low, mid, high, and top-level feature maps), the local feature extraction of the ResNet block is immediately enriched by the global contextual modeling of the MST block before being passed to the next stage.

Furthermore, unlike standard classification networks, this proposed architecture utilizes these enriched multi-scale features for a dual-purpose objective. The final feature maps are routed not only to a Classification Output branch but also to a dedicated Spatial Attention Module. This module generates spatial attention maps, effectively granting the model weak region localization capabilities without requiring computationally expensive, pixel-level bounding box annotations. Rather than serving as an autonomous diagnostic system, this model is designed to assist radiologists by highlighting potential nodules, thereby optimizing workflow efficiency and reducing the cognitive burden.

The main aim of this work is to develop a more sensitive computer-aided screening model that may help reduce false-negative interpretations in resource-constrained settings. Experimental results showed improved model performance relative to the baseline configuration. Most notably, the proposed RNNet-MST system achieved a mean Nodule Recall of 91.55 ± 1.41%, representing a 3.53% improvement over the baseline (88.02 ± 1.92%), alongside a mean Nodule F1-Score of 90.99 ± 0.39%, successfully outperforming the baseline architecture across key metrics.

While hybrid CNN–Transformer architectures have been widely explored in medical imaging, their application to pulmonary nodule analysis on chest X-rays remains relatively limited, particularly in resource-constrained screening settings. This study focuses on adapting such hybrid architectures specifically for CXR-based nodule detection, emphasizing sensitivity to small nodules and reduction in false-negative findings.

## 2. Materials and Methods

### 2.1. Dataset

This study utilized the NODE21 public dataset, a benchmark repository for pulmonary nodule detection on frontal-view chest radiographs. The dataset aggregates images from multiple sources, including JSRT, PadChest, ChestX-ray14, and Open-I, comprising 4882 images in total: 1134 positive cases containing 1476 annotated pulmonary nodules with radiologist-provided bounding boxes, and 3748 negative (nodule-free) cases. The dataset exhibits significant class imbalance, with the non-nodule class substantially overrepresented relative to the nodule class. All images were resized to 224 × 224 pixels to conform to the input layer specifications of the ResNet-50 backbone. The dataset was partitioned into training (70%), validation (15%), and test (15%) sets using a stratified patient-level split, with stratification by class label to ensure balanced class distribution across all partitions. Each unique patient appears exclusively in one partition, with zero patient-level overlap verified programmatically across all split pairs. Data augmentation was applied exclusively to the training set, while validation and test sets retained only original images.

### 2.2. Data Preprocessing and Augmentation

To improve generalization while preserving the intrinsic radiographic characteristics of chest X-ray images, controlled augmentation was applied only to the training set. The augmentation pipeline included resizing to 224 × 224 pixels, random horizontal flipping, small-angle rotation, mild translation, limited brightness/contrast perturbation, and normalization using the standard ImageNet mean and standard deviation. The nodule class was further upsampled to reduce class imbalance during training. PyTorch 2.6 + CUDA 12.4 DataLoader objects were configured with a batch size of 16 and num_workers = 4.

### 2.3. Baseline Model: ResNet-50

The baseline model is a ResNet-50 deep convolutional neural network pretrained on ImageNet [9]. ResNet-50 introduced residual learning through skip connections, enabling training of 50-layer networks without suffering from the vanishing gradient problem. Its final fully connected layer was replaced with a 2-class output layer (Nodule/No Nodule) for binary classification. The baseline was trained for 25 epochs using AdamW (lr = 1 × 10^−4^, weight decay = 0.01), a Cosine Annealing learning rate scheduler, and Cross-Entropy Loss.

Despite strong general classification capability, ResNet-50 has two documented limitations addressed in this study: (1) its local receptive fields cannot model long-range spatial dependencies, causing misclassification of smaller-diameter nodules; and (2) its ImageNet-pretrained weights are optimized for natural-image features that differ substantially from the subtle, texture-dependent patterns of pulmonary nodules on grayscale CXRs, resulting in reduced sensitivity on the medical domain.

### 2.4. Proposed Architecture: RNNet-MST

**Figure 1:**
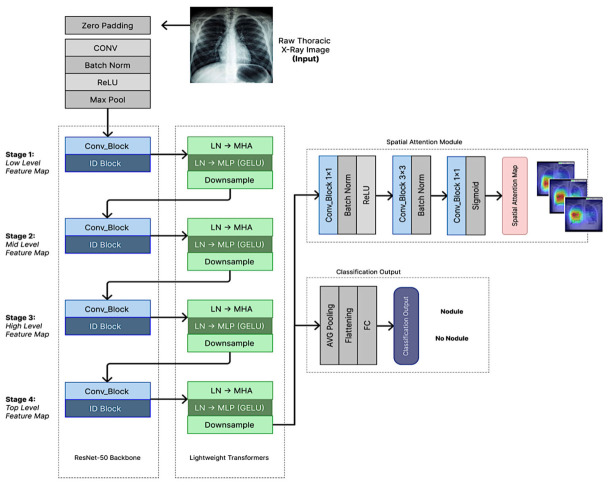
The proposed RNNet-MST architecture: MST blocks are integrated at all four stages of ResNet-50 to jointly capture long-range dependencies (Objective 1) and help mitigate the domain mismatch between ImageNet-pretrained features and CXR-specific patterns (Objective 2) in a single unified pipeline.

RNNet-MST extends the baseline ResNet-50 by hierarchically integrating Multi-Scale Transformer (MST) blocks across all four backbone stages (Figure 1). This design was intended to improve contextual feature modeling across scales and to better adapt pretrained convolutional features to grayscale chest radiographs. The resulting architecture combines convolutional feature extraction with transformer-based global context modeling in a single classification pipeline.

Each MST block utilizes 2 attention heads, an MLP ratio of 1.0, and a dropout rate of 0.1. To manage computational complexity while maintaining multi-scale depth, the block employs adaptive spatial downsampling; specifically, feature maps exceeding a resolution of 14 × 14 are pooled to a 14 × 14 grid before entering the attention mechanism, then bilinearly upsampled back to their original dimensions to maintain spatial consistency.

For final classification, a decision threshold of 0.5 is applied to the sigmoid output of the network. This threshold serves as the standard for distinguishing between positive and negative classes across all evaluated models in this study.

To capture long-range dependencies and global contextual information, MST blocks were hierarchically integrated at each of the four stages of the ResNet-50 backbone. Feature maps are extracted at four distinct stages: Stage 1—Low-Level Features; Stage 2—Mid-Level Features; Stage 3—High-Level Features; and Stage 4—Top-Level Features.

The output of each ResNet stage is passed into a corresponding Lightweight Transformer Block consisting of Layer Normalization (LN), Multi-Head Self-Attention (MHSA), and a Multi-Layer Perceptron (MLP) with GELU activation. Given an input image *I* ∈ R*^H^*^×^*^W^*^×^*^C^*, the feature map at stage *s* is produced by the ResNet stage function *f_s_*(·) applied to the output of the previous stage:

(1) *X_s_* = *f_s_*(*X_s_*_−1_), *X*_0_ = *I*

where *X_s_* ∈ R*^H^s* × *Ws* × *Cs* is the feature map at stage *s*, with *H_s_*, *W_s_*, and *C_s_* denoting its spatial height, width, and number of channels respectively. Each *X_s_* is then passed into a corresponding Lightweight Transformer block *T_s_*(·):

(2) *Y_s_* = *T_s_(X_s_)*

where *Y_s_* ∈ R*^H^s* × *Ws* × *Cs* is the transformer-enhanced feature map. To manage computational cost, *X_s_* is downsampled to 14 × 14 before the attention operation and upsampled back to *H_s_* × *W_s_* afterward. Let *S*_MLP_ ∈ R*^H^s* × *Ws* × *Cs* denote the output of the MLP sub-layer within *T_s_*(·), upsampled and reshaped back to the spatial dimensions of *X_s_*. The final output feature map *F*_out_ is then obtained by combining *S*_MLP_ with the original feature map *X_s_* via a residual connection to preserve low-level spatial information:

(3) *F*_out_ = *X_s_* + Upsample(reshape(*S*_MLP_))

where *F*_out_ ∈ R*^H^s* × *Ws* × *Cs* is the final enriched feature map passed to the next stage or classification head.

This hierarchical design ensures that global contextual information is captured continuously across all scales—from fine-grained textures in Stage 1 to high-level semantic structures in Stage 4—overcoming ResNet-50’s inherent local-receptive-field constraint.

In addition, the same MST integration simultaneously addresses the domain gap between ImageNet pretraining and medical radiographs. ResNet-50’s convolutional filters are optimized for the distinct edges and RGB textures of natural scenes, not the subtle grayscale texture patterns of pulmonary nodules on CXR. By applying self-attention across the entire CXR image at each feature scale, the transformer blocks enable the model to contextualize local convolutional features within the global thoracic structure, learning CXR-specific representations that compensate for ResNet-50’s natural-image inductive bias.

The MST blocks were integrated at all four stages to ensure that global contextual information is captured across multiple feature scales, from low-level textures to high-level semantic representations. Downsampling to 14 × 14 was used to balance computational efficiency with sufficient spatial resolution for attention modeling. Freezing the ResNet-50 backbone stabilizes training and preserves pretrained low-level feature representations, allowing the MST blocks to focus on adapting features to the target domain.

To further differentiate RNNet-MST from existing hybrid CNN–Transformer architectures such as ViT and Swin Transformer, three specific architectural distinctions are noted. First, rather than replacing the convolutional backbone entirely as in ViT or Swin, RNNet-MST hierarchically integrates lightweight transformer blocks at all four stages of a pretrained ResNet-50, preserving pretrained low-level feature representations while adapting higher-level features to the CXR domain. Second, the integration at all four stages ensures that global contextual information is captured continuously across multiple feature scales, from fine-grained textures in Stage 1 to high-level semantic structures in Stage 4, which is distinct from architectures that apply attention only at the final feature stage. Third, unlike general-purpose transformer architectures, RNNet-MST incorporates a custom spatial attention module inspired by multiplicative spatial attention mechanism of CBAM [14] that produces clinically interpretable localization maps aligned with radiologist-annotated nodule regions, which are used for weak localization analysis rather than dense detector-style supervision. This combination of hierarchical multi-scale transformer integration and spatially supervised attention constitutes thespecific architectural contribution of this work relative to existing hybrid approaches.

### 2.5. Training Configuration

To assess the stability and reproducibility of the models, all experiments were repeated across three independent runs using different random seeds (42, 444, and 916). Results are reported as mean ± standard deviation across runs. All experiments and model development were conducted using a personal computing system. The primary workstation was an Acer Nitro ANV15-51 laptop (Acer Inc., Xizhi, New Taipei City, Taiwan), equipped for deep learning model training and evaluation tasks.

During training, the ResNet-50 backbone weights (previously fine-tuned on NODE21) were frozen. Only the added MST blocks and the classification head were made trainable. The model was compiled with the following configuration:

- Optimizer: AdamW (*lr* = 1 × 10^−4^, weight decay = 0.01);
- Scheduler: Cosine Annealing Learning Rate;
- Loss Function: Weighted Binary Cross-Entropy (WBCE).

(4) $$L_{WBCE}=-\frac{1}{N}\sum_{i=1}^{N}\left[w_{1}y_{i}\log\left({\hat{y}}_{i}\right)+w_{0}\left(1-y_{i}\right)\log\left(1-{\hat{y}}_{i}\right)\right]\;$$

where *y_i_* is the ground-truth label, ${\hat{y}}_{i}$ is the predicted probability, and *w*_1_, *w*_0_ are class weights computed as:

(5) $$w_{C}=\frac{N}{\left|C\right|\times n_{C}}$$

yielding *w*_1_ = 1.7684 for the Nodule class and *w*_0_ = 0.6971 for the Non-Nodule class. Training ran for 25 epochs with a batch size of 16, saving model checkpoints whenever the total validation loss improved.

Model selection was based on the lowest validation F1-score. For reproducibility, key training details include the hardware configuration, software library versions, random seed, and decision threshold used for positive-class prediction. In this study, results are reported from a fixed train–validation–test split.

### 2.6. Evaluation Metrics

Model performance was assessed using the following metrics:

- Precision: TP/(TP + FP);
- Nodule Recall (Sensitivity): TP/(TP + FN);
- Nodule F1-Score: 2 × (Precision × Nodule Recall)/(Precision + Nodule Recall).

Where TP is the true positive, TN is the true negative, FP is the false positive, and FN is the false negative. Performance metrics are reported as mean ± standard deviation across three independent runs with random seeds to ensure reproducibility and assess result stability.

**Figure 2:**
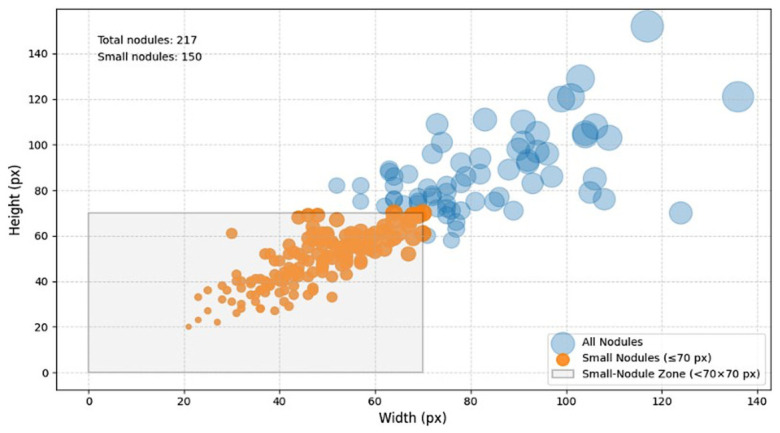
Distribution of BBox (width and height) within the selected small-nodule test subset, consisting of 171 CXR images with BBox width or height below 70 pixels.

To evaluate performance on small nodules, a dedicated small-nodule subset was isolated from the test set comprising 171 CXR images whose radiologist-annotated bounding boxes measured below 70 × 70 pixels in either dimension (Figure 2), targeting small and irregular nodules.

**Figure 3:**
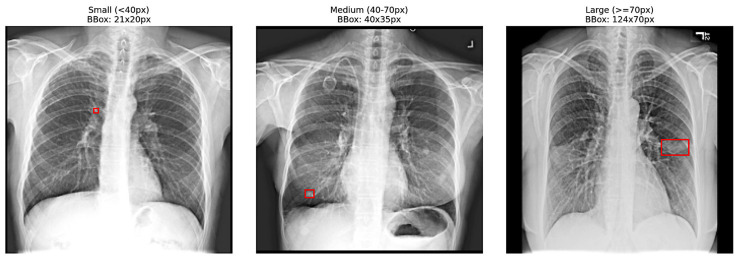
Visual comparison of attention heatmaps across varying nodule sizes. The red bounding boxes indicate the localized regions identifying the small, medium, and large pulmonary nodules within the lung fields.

To better visualize these cases, Figure 3 presents representative samples categorized by size. The subset isolated for this dedicated evaluation consists of the small and medium categories, where nodules are most susceptible to being overlooked. By isolating these 171 images for dedicated evaluation, we can better assess the model’s ability to localize subtle pathological features that are traditionally difficult to distinguish from complex anatomical noise in a standard chest radiograph.

Performance on this subset was measured by detection rate and false negative count. To evaluate Objective 2, Nodule Recall improvement was measured on the full test set, reflecting the reduction in domain-gap-related false negatives. Both evaluations use the same trained model per run—the distinction is analytical, not architectural; with results reported as mean ± standard deviation across three independent runs.

### 2.7. Comparative Statistical Evaluation

To rigorously assess whether the performance improvements of the proposed RNNet-MST architecture over the baseline ResNet-50 were statistically significant, McNemar’s test was utilized. Because both models were evaluated on the exact same test set of X-ray images, their predictions represent paired nominal data. McNemar’s test evaluates the discordant pairs—instances where one model predicted the nodule classification correctly while the other failed—to determine if the marginal frequencies are homogeneous.

To account for the discrete nature of the data and reduce Type I errors, Edwards’ continuity correction was applied. The test statistic is calculated as follows:

(6) $$X^{2}\;=\;\frac{{\left(\left|n_{10}\;-n_{01}\right|-\;1\right)}^{2}}{n_{10}\;+n_{01}}$$

where $n_{10}$ presents the number of images correctly classified by the RNNet-MST but misclassified by the baseline ResNet-50, and $n_{01}$ represents the number of images correctly classified by the baseline but misclassified by the RNNet-MST. A significance level $\left(\alpha \right)$ of 0.05 was established to reject the null hypothesis of equal model performance.

## 3. Results

### 3.1. General Classification Performance

**Figure 4:**
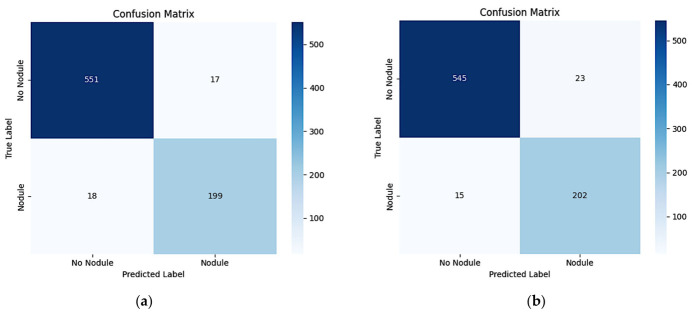
Test set classification performance confusion matrices. (**a**) Baseline ResNet-50. (**b**) Proposed RNNet-MST.

**Figure 5:**
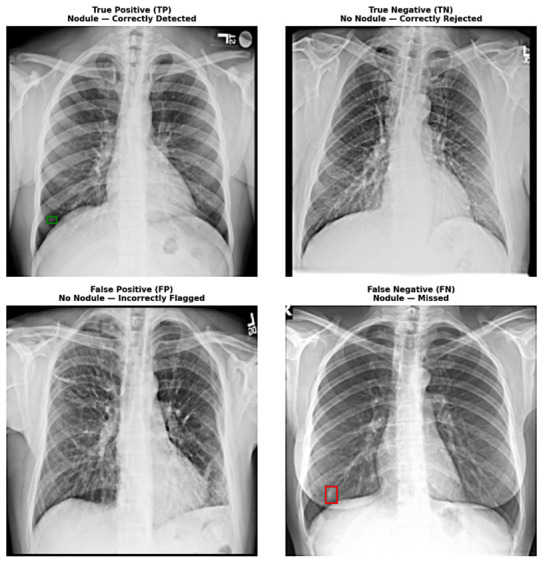
Representative examples of model classification: True Positive (TP), True Negative (TN), False Positive (FP), and False Negative (FN). The green bounding box denotes a correctly detected nodule (TP), while the red bounding box outlines a missed nodule (FN) that the model failed to detect.

Figure 4 presents the test set classification performance of the baseline ResNet-50 vs. RNNet-MST. Across three independent runs, the baseline ResNet-50 achieved a mean Nodule Recall of 88.02 ± 1.92%, indicating that approximately 12% of positive cases in the test set were missed on average. Following the integration of MST blocks, the RNNet-MST model achieved a mean Nodule Recall of 91.55 ± 1.41%, corresponding to consistently fewer false-negative classifications than the baseline model across all experimental runs. Figure 5 shows representative examples of model classification: True Positive (TP), True Negative (TN), False Positive (FP), and False Negative (FN). The green bounding box denotes a correctly detected nodule (TP), while the red bounding box outlines a missed nodule (FN) that the model failed to detect.

### 3.2. Comparative Evaluation

To evaluate the performance of RNNet-MST, we compared it against the baseline ResNet-50, a representative transformer backbone (Swin-T Small), and a strong CNN baseline (EfficientNet-B0). As shown in Table 1, while Swin Transformer achieved the highest raw performance in Nodule Recall (0.9378 ± 0.0122) and F1-score (0.9240 ± 0.0092), RNNet-MST demonstrated competitive and highly stable performance, achieving the lowest variance in F1-score (0.9099 ± 0.0039).

These results underscore a vital trade-off between peak accuracy and predictive consistency. Although the Swin Transformer achieves the highest recall, it also exhibits the highest variance, suggesting less stability across different data subsets. RNNet-MST addresses this by delivering the most stable performance, evidenced by an F1-score standard deviation of only 0.0039. This high level of reliability is particularly advantageous for clinical applications where consistent performance is paramount. By outperforming both ResNet-50 and EfficientNet-B0 in recall, RNNet-MST demonstrates that it can capture critical pathological features more effectively than traditional CNN baselines while maintaining superior robustness.

#### 3.2.1. Overall Classification Performance

As shown in Table 2, the integration of MST blocks improved performance across key metrics, though with an observed precision–recall trade-off. Nodule Recall increased from 88.02 ± 1.92% to 91.55 ± 1.41%, representing a mean absolute gain of 3.53%, directly reducing the rate of false-negative interpretations. Nodule F1-Score improved from 90.73 ± 1.52% to 90.99 ± 0.39%, with the reduced standard deviation indicating more consistent performance across runs. Test Accuracy improved from 94.94 ± 0.37% to 95.27 ± 0.28%.

While the proposed model achieves a marked improvement in Nodule Recall, Nodule Precision experienced a marginal decrease from 93.64 ± 2.13% to 90.46 ± 0.99%. We acknowledge that in a real-world clinical setting, this reduction in precision translates to a meaningful increase in false alarms. However, this reflects a deliberate and necessary trade-off in medical AI. In a lung cancer screening workflow, higher recall is prioritized [15], because false positives can be subsequently ruled out by a reviewing radiologist, whereas false negatives cannot be recovered and may severely delay life-saving diagnosis and treatment.

Consequently, the shift toward higher sensitivity—while maintaining manageable false positive rates—makes the proposed RNNet-MST model highly clinically viable as a primary triage tool. This clinical prioritization is firmly supported by Luo et al. [15], who emphasize that recall is the absolute most critical metric in pulmonary nodule detection; as a result, many computer-aided diagnosis (CAD) studies and screening guidelines favor maximizing sensitivity, even when it inherently increases the false positive rate.

The observed mean recall improvement of 3.53 ± 2.33% is consistent with the hypothesis that MST blocks improve contextual modeling and help adapt pretrained features to chest X-ray-specific patterns. Raghu et al. [16] demonstrated that ImageNet pretraining provides limited benefit for medical imaging tasks because visual feature distributions differ substantially between natural images and radiographs. By embedding MST blocks that apply self-attention over the image, the proposed model may better adapt ResNet-50 features to the visual characteristics of chest radiographs. This is further supported by Fu et al. [17], who demonstrated that hybrid CNN–Transformer architectures significantly outperform pure CNNs by overcoming spatial information loss and the style-over-content bias inherent in standard convolutional operations.

#### 3.2.2. Small-Nodule Detection Performance

To further assess the model’s sensitivity to the most diagnostically challenging cases, both models were additionally evaluated on an isolated small-nodule subset of 171 images whose radiologist-annotated bounding boxes measured below 70 × 70 pixels. As shown in Table 3, the baseline ResNet-50 correctly identified 138 nodules (80.7%) while failing to detect 33 cases (19.3%) as false negatives. RNNet-MST correctly classified 159 of 171 positive cases in the small-nodule subset, corresponding to a sensitivity of 93.0% and 12 false-negative cases (7.0%)—a 12.3% absolute improvement in sensitivity on the small-nodule subset.

On the full nodule test subset containing nodules of varying sizes (Table 4), the baseline ResNet-50 correctly identified 176 nodules (81.1%) and missed 41 cases (18.9%). RNNet-MST correctly classified 195 of 217 positive cases in the full nodule subset, corresponding to a sensitivity of 89.9% and 22 false-negative cases (10.1%)—an 8.8% improvement in sensitivity across heterogeneous nodule sizes.

These results suggest that the hierarchical MST integration helps mitigate the limitations of ResNet-50’s restricted receptive field by enabling broader contextual modeling across the CXR. This is consistent with Raghu et al. [18], who demonstrated that Vision Transformer layers preserve spatial location information more effectively than ResNet layers, and with Dai and Gao [19], who showed that hybrid CNN–Transformer models consistently outperform pure CNNs in medical image classification.

All experiments were conducted using a fixed train–validation–test split and repeated across three independent runs to assess performance variability and robustness.

#### 3.2.3. Computational Efficiency and Inference Latency

To evaluate the clinical feasibility of the proposed RNNet-MST, we benchmarked its computational requirements and inference latency against the ResNet-50 baseline. Table 5 summarizes the total parameter count and the average execution time across both GPU and CPU environments. Benchmarking was conducted using an NVIDIA GeForce RTX 4060 Laptop GPU and an Intel Core i5-13420H CPU. To ensure statistical reliability, timings were recorded over three independent experiments, each consisting of 100 inference runs following 10 warmup iterations.

The results indicate that the integration of the Multi-Scale Transformer and spatial attention modules increases the parameter count to approximately 57 million. The proposed model achieves a mean inference time of 11.70 ms/image, representing a manageable increase of approximately 4.04 ms over the baseline. While the CPU latency is higher at 110.94 ms/image, it remains well within the acceptable threshold for real-time diagnostic workflows. In a clinical setting, a total processing time of roughly 0.1 s per scan is virtually imperceptible to the user.

### 3.3. Ablation Study

#### 3.3.1. Ablation Study of MST Block Integration Across ResNet-50 Stages

To systematically assess the necessity and individual contribution of the Multi-Scale Transformer (MST) blocks, an ablation study was conducted evaluating partial network integrations against the baseline and the proposed full architecture. Specifically, models were trained with MST blocks integrated only at early stages (Stages 1–2) and only at late stages (Stages 3–4). To ensure statistical reliability and assess model stability, all configurations were evaluated across multiple random weight initialization seeds, with results reported as the mean and standard deviation (Mean ± SD).

As presented in Table 6, the partial integration of MST blocks fails to achieve the optimal local-global feature synergy required for robust nodule detection. For the critical “Nodule” class, integrating blocks exclusively at late stages (Stages 3–4) resulted in a decrease in overall performance, yielding a mean F1-Score of 0.8641 ± 0.0396, which underperforms the ResNet-50 baseline (0.9073 ± 0.0152). While early-stage integration (Stages 1–2) demonstrated slight improvements over late-stage integration, it still exhibited high variance (± 0.0481 for Nodule Recall) and failed to surpass the baseline’s classification balance.

Conversely, the proposed full integration across all four stages (ResNet-50 + MST Stages 1–4) achieved the highest overall diagnostic performance for the positive class. The full architecture maximized the mean Nodule Recall to 0.9155, effectively minimizing false negatives. Furthermore, integrating all four MST blocks significantly stabilized the model’s predictive capability across different initializations, reducing the standard deviation of the Nodule F1-Score to a mere ± 0.0039. These results empirically validate the proposed architectural design, demonstrating that deep, stage-wise integration across all four levels is essential for achieving both high sensitivity and consistent, reproducible performance.

#### 3.3.2. Evaluation of the Spatial Attention Mechanism on Localization Metrics

Beyond general classification, it is necessary to empirically isolate the mechanistic contributions of the multi-scale contextual modeling and the spatial attention module. To achieve this, an ablation study was conducted to evaluate the network’s ability to localize disease-relevant regions. The isolated components—specifically the baseline ResNet-50, ResNet-50 with Spatial Attention only, RNNet-MST (without attention), and the full RNNet-MST with Spatial Attention—were evaluated across BBox Coverage, Detection Rate, Peak Proximity, and Attention Focus. Results are reported as the mean and standard deviation (Mean ± SD) across multiple initializations.

As demonstrated in Table 7, standard convolutional architectures struggle with weak localization. The ResNet-50 baseline yielded a poor mean Detection Rate of 0.2949 and exhibited high variance (±0.1605). Interestingly, directly appending the Spatial Attention Module to the standard ResNet-50 backbone resulted in a marginal degradation of localization performance, dropping the BBox Coverage to 0.2941. This indicates that the localized feature maps of a standard CNN lack the rich global context required for the attention mechanism to effectively isolate subtle nodules.

However, the proposed full architecture (RNNet-MST + Spatial Attention) demonstrates a synergistic effect. By leveraging the globally enriched feature maps generated by the MST blocks, the Spatial Attention Module is able to function optimally. This combination achieves a superior mean BBox Coverage of 0.4955 and nearly doubles the baseline’s Detection Rate to 0.5695. Furthermore, the exceptionally low standard deviations (e.g., ±0.0181 for BBox Coverage) confirm that this dual-branch design consistently and reliably guides the network to focus on true nodule locations, validating its effectiveness as a diagnostic aid. Additional qualitative attention map examples and quantitative localization analyses are provided in Appendix A.

### 3.4. Statistical Significance of Model Performance

A head-to-head comparative analysis of the baseline ResNet-50 and the proposed RNNet-MST was conducted on the complete test set, comprising 785 images. Out of the total predictions, the models were concordant on 729 cases, with 713 instances where both models predicted correctly and 16 instances where both failed. The statistical evaluation focused specifically on the 56 discordant cases, as shown in Table 8.

The baseline ResNet-50 correctly classified 19 instances that the proposed model missed ($n_{01}$= 19). Conversely, the proposed RNNet-MST successfully classified 37 instances that the baseline model failed to detect ($n_{10}$ = 37), demonstrating a clear reduction in classification errors. Application of McNemar’s test with continuity correction to these discordant pairs yielded a Chi-Square statistic of 5.1607 and a corresponding *p*-value of 0.0231.

**Table 8:** Contingency Table for McNemar’s Test on the Test Set (*n* = 785).

Models	RNNet-MST Correct	RNNet-MST Incorrect
ResNet-50 Correct	713	19
ResNet-50 Incorrect	37	16

McNemar’s test with continuity correction yielded $X^{2}$ = 5.1607 and *p* = 0.02310, indicating a statistically significant difference in performance (*p* < 0.05).

Because the *p*-value is strictly less than the predefined significance threshold (*p* < 0.05), the null hypothesis is rejected. This confirms that the integration of the multi-scale transformer blocks in the RNNet-MST provides a statistically significant improvement in predictive accuracy over the standard ResNet-50 baseline.

## 4. Discussion

### 4.1. Interpretation of Performance Gains

The primary objective of this study was to address the critical diagnostic gap in pulmonary nodule detection, characterized by high rates of false-negative interpretations due to the anatomical complexity of CXRs and radiologist fatigue. The proposed RNNet-MST architecture improved Nodule Recall by 3.53 percentage points relative to the baseline ResNet-50 model.

These findings are consistent with the view that standard convolutional neural networks are limited by local receptive fields when subtle abnormalities must be interpreted within broader anatomical context. By integrating Multi-Scale Transformer (MST) blocks, the proposed model effectively captured the long-range spatial dependencies required to contextualize subtle radiographic abnormalities. This improvement was most pronounced in the targeted small-nodule subset, where the RNNet-MST model increased the detection rate by 12.3% and reduced false negatives from 33 to just 12 cases. These results align with Raghu et al. [18], who demonstrated that Vision Transformers preserve spatial location information more effectively than pure CNNs, and corroborate the findings of Fu et al. [17] regarding the superiority of hybrid architectures in overcoming CNN style-over-content biases in medical imaging.

### 4.2. Comparison with Related CXR-Based Nodule Detection Work

To situate the performance of RNNet-MST within the broader literature, Table 9 summarizes representative deep learning methods for pulmonary nodule detection on chest radiographs. A consistent observation across the surveyed works is that CXR-based detection methods report substantially lower sensitivity benchmarks than their CT counterparts, underscoring the inherent difficulty of the task and the importance of continued research on this modality. Behrendt et al. [20] achieved the highest reported sensitivity in the CXR domain through an ensemble of four state-of-the-art object detectors trained on the same NODE21 dataset, winning the Node21 challenge; however, their approach carries a substantially higher computational cost due to the multi-model ensemble strategy. Against this backdrop, RNNet-MST achieved a Nodule Recall of 91.55% on the NODE21 test set, representing one of the strongest recall values among the CXR-based methods summarized here, although direct cross-study comparison remains limited by differences in datasets, task definitions, and evaluation protocols. Extended comparative benchmarking details are provided in Appendix B.

### 4.3. Clinical Significance of the Precision–Recall Trade-Off

A critical point of interpretation in this study is the observed precision–recall trade-off. While Nodule Recall improved significantly to 91.55%, Nodule Precision decreased from 0.94 to 0.90. We acknowledge that in a real-world screening workflow, this reduction in precision is not trivial; it translates directly to a meaningful increase in false alarms, which inevitably adds to the radiologist’s cognitive load as they must visually review and dismiss these non-malignant regions.

However, the clinical acceptability of this trade-off must be evaluated concretely within the model’s intended deployment setting: serving as an automated second-reader triage tool in high-volume, resource-constrained environments. As emphasized by Luo et al. [15], prioritizing sensitivity is paramount in cancer screening because the clinical penalties are highly asymmetrical. The clinical “cost” of a false positive is the additional seconds required for a radiologist to overrule the CAD system or, at worst, the scheduling of a secondary CT scan. Conversely, the cost of a false negative—a missed malignant nodule—is a severely delayed diagnosis that can prove fatal.

Conversely, maintaining a high baseline of precision remains vital to the practical success of any Computer-Aided Detection (CAD) system. If a model’s precision falls too low, the resulting influx of false positives can induce “alarm fatigue,” a phenomenon where overwhelmed clinicians become desensitized to automated alerts, thereby neutralizing the tool’s clinical value. Furthermore, excessive false alarms can trigger unwarranted psychological distress for patients and lead to unnecessary, resource-intensive follow-up imaging. Therefore, while the RNNet-MST sacrifices a fraction of its precision for a vital gain in sensitivity, maintaining a robust precision score of 0.90 ensures that the system does not overwhelm the diagnostic workflow with spurious findings.

Because RNNet-MST is explicitly designed to mitigate the diagnostic blind spots and perceptual errors made by fatigued radiologists interpreting hundreds of scans daily [4,5], prioritizing a 91.55% recall rate represents a deliberate and clinically sound compromise. By flagging a broader set of suspicious regions while maintaining manageable false positive rates, the model successfully minimizes the risk of missed nodules on first-line chest radiographs, successfully fulfilling its primary clinical objective as a highly viable triage tool.

### 4.4. Workflow Optimization in Resource-Constrained Settings

Furthermore, these performance gains carry significant implications for the Philippine healthcare system. Expert interviews conducted at the Lung Center of the Philippines highlighted that radiologists routinely interpret up to 200 images daily, leading to intense cognitive load. By compensating for the domain gap between natural-image pretraining and grayscale radiographs, RNNet-MST provides attention maps that may support sensitive visual assessment of suspicious regions. This functions not as an autonomous diagnostic replacement, but as an intelligent visual aid designed to reduce perceptual errors, streamline workflow, and alleviate professional burnout in resource-constrained environments.

It is important to note that the proposed system operates as a classification-based computer-aided detection (CAD) model with weak localization via attention maps, rather than a fully supervised object detection framework. While object detection models such as Faster R-CNN or DETR provide explicit bounding box predictions, they typically require dense annotations and higher computational resources. In contrast, the proposed approach prioritizes computational efficiency relative to full object-detection frameworks while still providing clinically useful localization guidance for screening applications in resource-constrained clinical environments. Additional pre-clinical assessment details and supporting analyses are provided in Appendix C.

### 4.5. Study Limitations and Future Research Directions

This study has several limitations. First, the model was assessed on a single public dataset, which limits conclusions about generalizability across institutions and acquisition settings. Second, the localization analysis was attention-based and therefore should be interpreted as limited localization guidance rather than detector-level lesion localization. More established localization endpoints such as pointing game accuracy, center-hit rate, and thresholded IoU were not employed, as these metrics are designed for dedicated object detection frameworks with explicit localization outputs. Third, the dataset primarily consists of frontal-view radiographs and may not fully capture the variability present in real-world clinical settings, particularly in local Philippine hospitals. Fourth, the current architecture is also limited to binary classification (nodule vs. no nodule) and does not differentiate between benign and malignant nodules, which restricts its direct clinical interpretability. Fifth, source labels are not available in the publicly released NODE21 dataset, precluding source-stratified analysis or leave-one-source-out validation. Future work will prioritize datasets with explicit source annotations to enable more rigorous evaluation of generalization across acquisition settings.

Future research directions include extending the proposed hybrid backbone into object detection frameworks, such as Faster R-CNN or DETR, to enable precise bounding box prediction rather than relying on classification and attention-based localization. Additionally, incorporating histopathologically confirmed datasets would allow the model to perform multi-class malignancy classification. Finally, validation on larger, independent clinical cohorts—particularly using localized data from Philippine medical institutions—will be essential to ensure clinical robustness and deployment readiness.

## 5. Conclusions

This study proposed RNNet-MST, a hybrid deep learning architecture for pulmonary nodule classification on chest X-ray (CXR) images, developed by enhancing ResNet-50 with Multi-Scale Transformer (MST) blocks. The proposed model was designed to address two documented limitations of the baseline ResNet-50: its limited ability to capture long-range dependencies relevant to small-nodule classification, and its reduced sensitivity associated with the domain gap between ImageNet pretraining and CXR-specific features.

Experimental results on the NODE21 dataset showed consistent improvements across the primary evaluation metrics across three independent runs. Most critically, the model achieved a mean Nodule Recall of 91.55 ± 1.41%, representing a 3.53% improvement over the baseline (88.02 ± 1.92%), corresponding to fewer false-negative classifications. Mean Nodule F1-Score improved from 90.73 ± 1.52% to 90.99 ± 0.39%, with the reduced standard deviation indicating more stable performance across runs. On the isolated small-nodule subset, the proposed model achieved a 12.3% improvement in sensitivity over the baseline.

These findings suggest that combining convolutional feature extraction with multi-scale transformer-based contextual modeling can improve sensitivity in CXR-based pulmonary nodule classification. This may be particularly valuable in resource-constrained clinical settings, where high radiologist workloads and limited access to CT imaging increase the need for assistive screening tools. Future work should extend this architecture toward full bounding box detection using frameworks such as Faster R-CNN or DETR, incorporate benign-to-malignant nodule classification using histopathologically confirmed datasets, and validate the model across additional large-scale and independent datasets to strengthen generalizability.

## Figures and Tables

**Table 1:** Performance metrics comparison of RNNet-MST against other models on the same NODE21 test set split.

Model	Nodule Precision	Nodule Recall	Nodule F1-Score
ResNet-50	0.9364 ± 0.0213	0.8802 ± 0.0192	0.9073 ± 0.0152
Swin Transformer	0.9110 ± 0.0265	0.9378 ± 0.0122	0.9240 ± 0.0092
EfficientNet	0.9256 ± 0.0201	0.8595 ± 0.0201	0.8913 ± 0.0183
RNNet- MST (Present Study)	0.9046 ± 0.0099	0.9155 ± 0.0141	0.9099 ± 0.0039

**Table 2:** Per-class classification performance of baseline ResNet-50 and proposed RNNet-MST on the NODE21 test set.

Metric	Class	Precision	Recall	F1-Score
ResNet-50	No Nodule	0.9553 ± 0.0069	0.9772 ± 0.0084	0.9661 ± 0.0057
	Nodule	0.9364 ± 0.0213	0.8802 ± 0.0192	0.9073 ± 0.0152
RNNet-MST	No Nodule	0.9676 ± 0.0051	0.9630 ± 0.0046	0.9653 ± 0.0013
	Nodule	0.9046 ± 0.0099	0.9155 ± 0.0141	0.9099 ± 0.0039

**Table 3:** Comparative analysis of baseline ResNet-50 vs. RNNet-MST on the small-nodule subset (≤70 × 70 px bounding boxes).

Model	Correctly Detected	False Negatives	Detection Rate
Baseline ResNet-50	138/171	33	80.7%
RNNet-MST	159/171	12	93.0%

**Table 4:** Comparative analysis of baseline ResNet-50 vs. RNNet-MST on nodules of varying sizes.

Model	Correctly Detected	False Negatives	Detection Rate
Baseline ResNet-50	176/217	41	81.1%
RNNet-MST	195/217	22	89.9%

**Table 5:** Comparison of GPU & CPU computational cost and inference latency (Mean ± SD).

Model	Total Parameters	GPU Inference (ms)	CPU Inference (ms)
Baseline ResNet-50	23,512,130	7.66 ± 0.42	69.49 ± 2.51
RNNet-MST	56,973,890	11.70 ± 0.56	110.94 ± 0.95

**Table 6:** Standard deviation performance metrics for the ablation study of MST block integration across different ResNet-50 stages.

Model	Class	Precision	Recall	F1-Score
Resnet-50 Baseline	No Nodule	0.9553 ± 0.0069	0.9772 ± 0.0084	0.9661 ± 0.0057
	Nodule	0.9364 ± 0.0213	0.8802 ± 0.0192	0.9073 ± 0.0152
Resnet-50 + MST Stages 1–2	No Nodule	0.9665 ± 0.0170	0.9507 ± 0.0208	0.9583 ± 0.0029
	Nodule	0.8788 ± 0.0421	0.9124 ± 0.0481	0.8938 ± 0.0056
Resnet-50 + MST Stages 3–4	No Nodule	0.9484 ± 0.0157	0.9478 ± 0.0148	0.9481 ± 0.0150
	Nodule	0.8635 ± 0.0387	0.8648 ± 0.0415	0.8641 ± 0.0396
Resnet-50 + MST Stages 1–4	No Nodule	0.9676 ± 0.0051	0.9630 ± 0.0046	0.9653 ± 0.0013
	Nodule	0.9046 ± 0.0099	0.9155 ± 0.0141	0.9099 ± 0.0039

**Table 7:** Performance Comparison Across Ablation Configurations.

Model	Bbox Coverage	Detection Rate	Peak Proximity	Attention Focus
ResNet-50 Baseline	0.3106 ± 0.1045	0.2949 ± 0.1605	0.7798 ± 0.0279	2.6193 ± 0.4200
ResNet-50 + Spatial Attention	0.2941 ± 0.0932	0.2786 ± 0.1028	0.7907 ± 0.0412	3.1912 ± 0.0939
RNNet-MST	0.3364 ± 0.0093	0.2691 ± 0.0182	0.7649 ± 0.0148	1.6657 ± 0.0847
RNNet-MST + Spatial Attention	0.4955 ± 0.0181	0.5695 ± 0.0391	0.7641 ± 0.0245	2.5845 ± 0.1326

**Table 9:** Comparison of RNNet-MST with related deep learning methods for pulmonary nodule detection on chest radiographs (CXR).

Study	Year	Method/Model	Dataset	Sensitivity/ Recall	Key Notes
Yoo et al. [21]	2020	Deep learning algorithm (commercial CAD)	NLST	74.0%	5485 participants; specificity 73%; ***AUC 0.86
Schultheiss et al. [6]	2021	RetinaNet/U-Net CNN	Synthetic (from LIDC-IDRI CT)	**wAFROC: 0.81	201 synthetic radiographs; *p* = 0.49 vs. radiologists
Chiu et al. [8]	2022	YOLOv4 + U-Net lung segmentation	TVGH + JSRT	79.0%	3.04 FP/image; 254 CXRs tested
Shimazaki et al. [7]	2022	CNN segmentationbased DL model	In-house (Osaka City Univ.)	73.0%	0.13 mFPI; lower sensitivity in blind spots (50–64%)
Behrendt et al. [20]	2023	Ensemble (Faster- RCNN, RetinaNet, EfficientDet-D2, YOLOv5)	NODE21	*FROC25%: ∼0.84	Node21 competition winner; AUROC + FROC metric
**Present study**	**2026**	**RNNet-MST**	**NODE21**	**91.55%**	**Reports strong recall relative to the CXR-based methods summarized here**

*FROC_25%_ = sensitivity at 25% false positive rate; **wAFROC = weighted alternative free-response ROC figure of merit; ***AUC = area under the ROC curve. Note: Direct comparison across studies should be interpreted cautiously due to differences in datasets, evaluation protocols, and metric definitions.

**Figure A1:**
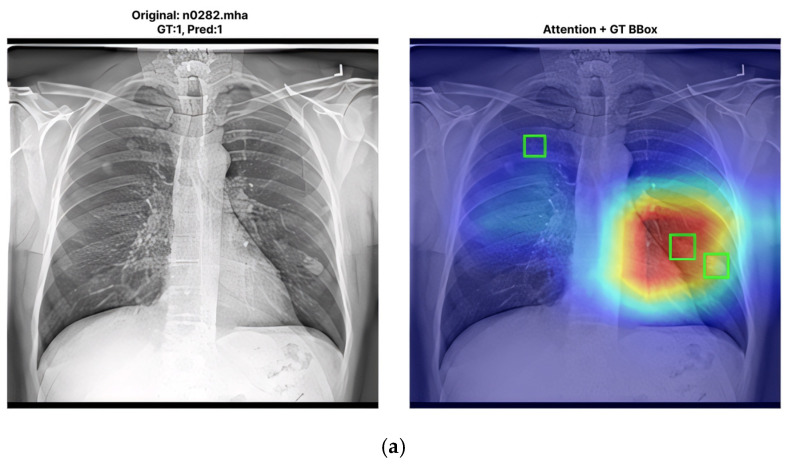

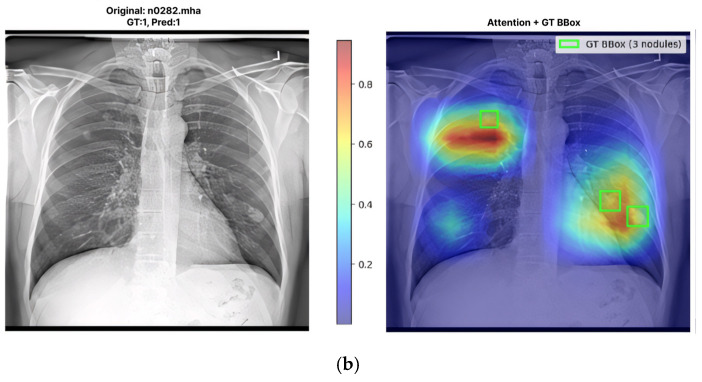
Heatmap Activation comparison of (**a**) Baseline vs. (**b**) Enhanced Model attention for patient n0282 in the test subset derived from the NODE21 dataset.

**Figure A2:**
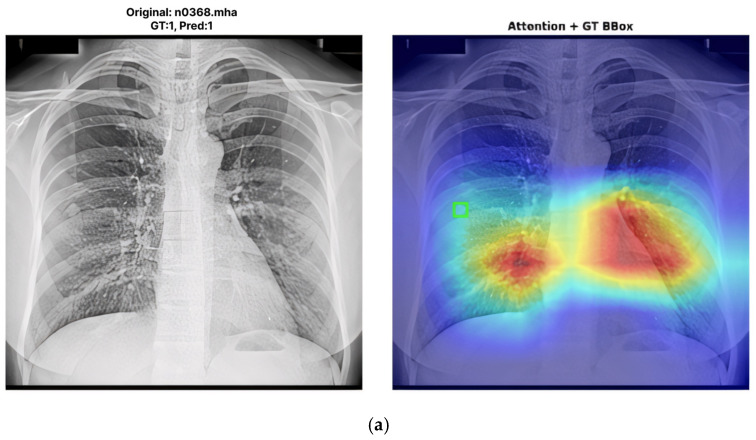

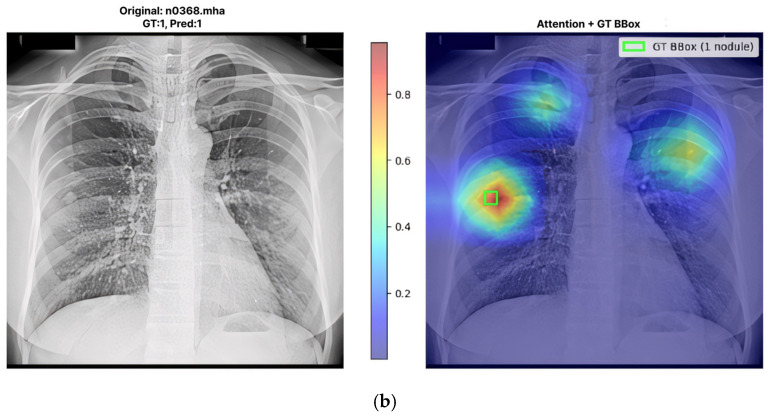
Heatmap Activation comparison of (**a**) Baseline vs. (**b**) Enhanced Model attention for patient n0282 in the test subset derived from the NODE21 dataset. The green boxes indicate the ground truth (GT) bounding box coordinates of nodule from the NODE21 metadata.

**Figure A3:**
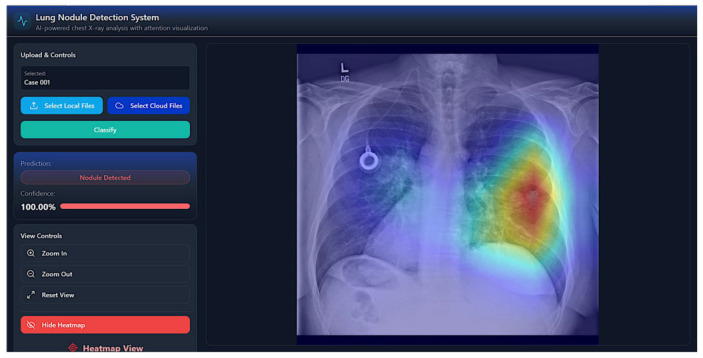
The system interface integrated with the enhanced spatial attention model for radiologist evaluation.

## Data Availability

The dataset used in this study is the publicly available NODE21 chest X-ray dataset. Dataset Link: https://zenodo.org/records/5548363; Date of Access: 3 November 2025. Additional implementation details and derived materials are available from the corresponding authors.

## Abbreviations

The following abbreviations are used in this manuscript:

CAD	Computer-Aided Detection
CBAM	Convolutional Block Attention Module
CNN	Convolutional Neural Network
CT	Computed Tomography
CXR	Chest X-Ray
FC	Fully Connected
FN	False Negative
FP	False Positive
GELU	Gaussian Error Linear Unit
LCP	Lung Center of the Philippines
LN	Layer Normalization
MHSA	Multi-Head Self-Attention
MLP	Multi-Layer Perceptron
MST	Multi-Scale Transformers
NODE21	Nodule Detection 2021
ResNet	Residual Neural Network
RNNet-MST	Residual Neural Network with Multi-Scale Transformers
TN	True Negative
TP	True Positive
ViT	Vision Transformer
WBCE	Weighted Binary Cross-Entropy

## Appendix A. Quantitative and Qualitative Analysis of Spatial Attention

### Appendix A.1. Quantitative Analysis of Baseline vs. Enhanced Model

As RNNet-MST is designed as a classification-based screening model with attention-guided localization, the following analysis evaluates the degree to which the learned attention maps correlate with annotated nodule regions rather than serving as a fully supervised object-detection evaluation. The attention localization performance was evaluated using four complementary metrics designed to assess whether nodule bounding boxes fall within high-attention regions, which is a critical requirement for assistive/computer-aided diagnostic systems.

BBox Coverage measures the average attention intensity within ground truth nodule regions (0–1 scale), with higher values indicating stronger highlighting of pathological areas. Detection Rate quantifies the percentage of nodules that fall within high-attention regions (threshold > 0.5), providing a binary measure of localization success. Peak Proximity evaluates the normalized distance from the maximum attention point to the nodule center (0–1 scale), where scores approaching 1.0 indicate precise centering of attention on nodules. Attention Focus Ratio compares the mean attention inside versus outside nodule regions, with values >1.0 indicating preferential focus on pathological tissue; this metric is particularly important for reducing false highlights in clinical settings.

As shown in Table A1, the enhanced RNNet-MST + Spatial Attention model demonstrates consistent and substantial improvements across primary localization metrics. A key observation is the synergy between the Multi-Scale Transformer (MST) and the spatial attention module. While the ResNet-50 + Spatial Attention model achieves the highest Attention Focus (3.1912 ± 0.0939), it does not yield a corresponding gain in Bbox Coverage. Conversely, the RNNet-MST alone produces a lower focus ratio at 1.6657 ± 0.0847. This indicates that the transformer’s global attention mechanism tends to spread across the entire image to capture contextual features for classification rather than concentrating specifically on the nodules. Only the combined architecture leverages the rich contextual features of the MST to allow the spatial attention module to concentrate on relevant regions, resulting in a dramatic increase in Bbox Coverage to 0.4955 ± 0.0181 and a Detection Rate of 0.5695 ± 0.0391.

**Table A1:** Attention-bounding box alignment performance metrics for baseline ResNet-50 with Grad-CAM and enhanced RNNet-MST with the spatial attention module.

Model	Bbox Coverage	Detection Rate	Peak Proximity	Attention Focus
ResNet-50 + Spatial Attention	0.2941 ± 0.0932	0.2786 ± 0.1028	0.7907 ± 0.0412	3.1912 ± 0.0939
RNNet-MST + Spatial Attention	0.4955 ± 0.0181	0.5695 ± 0.0391	0.7641 ± 0.0245	2.5845 ± 0.1326

These results further highlight the tradeoff between localization and classification. While the RNNet-MST achieves high classification performance, its relatively modest localization metrics confirm that these two tasks are distinct in a weakly supervised setting. The model can correctly classify nodules without necessarily producing concentrated heatmaps. The addition of spatial attention effectively bridges this gap by focusing computational resources on clinically relevant pathological regions.

It is acknowledged that more established localization endpoints such as pointing game accuracy, center-hit rate, and thresholded IoU were not employed, as these metrics are designed for dedicated object detection frameworks with explicit localization outputs. The metrics reported here were selected to be appropriate for the weakly supervised, classification-first nature of the proposed model.

### Appendix A.2. Visual Comparison of Baseline vs. Enhanced Model

The resulting heatmaps showed that the model consistently focused on anatomically relevant regions, particularly the lungs, indicating more reliable and clinically meaningful localization. Qualitative analysis of representative cases revealed distinct differences in attention behavior between the baseline and enhanced models.

Figure A1 presents a particularly notable case involving three nodules (Patient n0282), two in the right lung and one in the left lung. The baseline model captured only the two nodules in the right lung, completely missing the solitary left lung nodule. In contrast, the enhanced model demonstrated attention coverage across all three nodules, suggesting that the multi-scale light transformers embedded in the enhanced ResNet-50 model contribute to improved spatial awareness through long-range dependency modeling and global context aggregation. This ability to simultaneously attend to spatially distant pathological regions indicates that the transformer components enable the model to integrate information across the entire lung field rather than focusing exclusively on locally salient features.

Furthermore, Figure A2 (Patient n0368) demonstrates distributed attention across different lung regions while maintaining peak activation precisely within the bounding box of a very small nodule in the right lung. This behavior suggests that the multi-scale light transformer architecture influences attention allocation decisions by balancing global context awareness with fine-grained local feature discrimination, enabling detection of subtle pathological findings that might otherwise be overlooked in traditional convolutional approaches.

## Appendix B. Techniques to Address Class Imbalance

To ensure the model remains highly sensitive to pathological findings, specific data augmentation and loss weighting strategies were implemented to address the severe class imbalance and eliminate the inherent bias toward the majority “No Nodule” class.

Since the dataset was heavily imbalanced, the researchers applied data augmentation techniques to the training set. Only minimal augmentation was performed to preserve the intrinsic characteristics of the chest X-rays:

- Horizontal Translation: Random shifts of ±2% of image width.
- Vertical Translation: Random shifts of ±2% of image height.
- Rotation: Small random rotations within ±3 degrees.
- Brightness Adjustment: Multiplicative intensity scaling by random factor between 0.95–1.05 (±5%), clipped to original intensity range.
- Horizontal Flip: Left-right mirroring of the image.
- Combined Transform: Horizontal shift (±1%) combined with rotation (±2°).

However, during the training of the spatial attention layer, augmented images from the upsampled set could not be paired with accurate bounding box coordinates due to random shifts, flips, and rotations. Due to this, the researchers could only utilize the original unbalanced dataset for the training of spatial attention layers.

To address this limitation, the researchers used Weighted Binary Cross-Entropy (WBCE) to compensate for the imbalance when training the spatial attention layer without reliable bounding box information. The class weights were computed using the formula: class weights = total samples/(len (class_counts) x class counts).

This weighting strategy helped the model remain sensitive to true nodules even in the absence of spatial labels. The final calculated weights were 1.7684 for the Nodule class and 0.6971 for the Non-Nodule class.

## Appendix C. Preliminary Clinical Interface Evaluation and Directions for Future Validation

To obtain initial qualitative insights into the clinical utility and interface usability of the proposed system, a formative pilot evaluation was conducted with a single practicing radiologist from the College of Medicine at the Pamantasan ng Lungsod ng Maynila (PLM) with 6 years of clinical experience. The radiologist interacted with the system interface integrating the proposed AI tool (Figure A3) and completed a structured survey across three domains: Diagnostic Alignment and Accuracy, Clinical Impact and Decision Making, and System Usability, rated on a 5-point scale.

System usability received the highest scores (5/5 across all usability metrics), suggesting that the interface design—including heatmap visualization and zoom functionality—is intuitive and ready for broader clinical testing. Diagnostic alignment and automated classification agreement received neutral scores (3/5), while the utility of the AI in identifying subtle nodules in cases of discrepancy was rated lower (2/5). Rather than undermining the proposed model, these scores honestly reflect the current performance ceiling of a single-dataset, classification-based system and directly motivate the directions for future work outlined in Section 4.5. Importantly, despite the neutral alignment scores, the radiologist reported that the AI output prompted reconsideration or refinement of the final diagnosis (4/5) and increased confidence in the final diagnosis after using the tool (4/5), suggesting potential clinical value as a second-opinion aid even at this early stage.

The primary strength noted qualitatively was system usability, described as user friendly and easy to navigate. The key area for improvement identified was the need for training on more diverse samples to improve nodule detection precision—consistent with the generalizability limitations discussed in Section 4.5.

These preliminary findings should be interpreted as formative and exploratory, intended to identify early interface and performance trends rather than provide statistically generalizable clinical conclusions. They directly inform the design of a planned prospective validation study involving a larger cohort of radiologists across multiple Philippine clinical institutions, incorporating standardized usability instruments such as the System Usability Scale (SUS) and blinded diagnostic comparison against radiologist ground truth. Full clinical validation, including multi-reader agreement analysis and evaluation on locally acquired Philippine hospital data, is reserved as a dedicated subsequent study.
